# Unveiling the non-canonical functions of EZH2 in prostate cancer

**DOI:** 10.18632/oncotarget.28357

**Published:** 2023-02-11

**Authors:** Yang Yi, Yanqiang Li, Kaifu Chen, Qi Cao

**Keywords:** EZH2, prostate cancer, FBL, CDCA8, E2F1

Prostate cancer (PCa) is ranked as the second leading cause of cancer-related death among American men excluding skin cancer [1]. As a well-known oncogenic driver in PCa, enhancer of zeste homolog 2 (EZH2) is canonically known for the functions as the catalytic subunit of Polycomb Repressive Complex 2 (PRC2) that deposes histone H3 lysine 27 mono, di-, and tri-methylation (H3K27me1-3) and represses transcription [2, 3]. Although the oncogenic role of EZH2 mainly relies on its enzymatic activity and the PRC2, accumulating evidence suggests that targeting the lysine methyltransferase activity of EZH2 alone is ineffective in treating EZH2-dependent malignancies including PCa [4, 5]. Hence, deeply investigating the multifaceted tumorigenic functions of EZH2 will shed new light on understanding the etiology of PCa. It is noteworthy that two recent studies published in *Nature Cell Biology* and *Oncogene* by Yi et al. described previously unrecognized roles of EZH2 in regulation of translation [6] and co-activation of transcription [7], respectively. In both cases, EZH2 exerts oncogenic functions independently of PRC2 and H3K27me3 to promote tumorigenesis and aggressiveness in PCa.

Yi et al. observed a direct interaction between EZH2 and rRNA methyltransferase fibrillarin (FBL) in the nucleus of PCa cells [6]. By forming the box C/D small nucleolar ribonucleoprotein (box C/D snoRNP) with a guiding small nucleolar RNA as well as three other protein components (NOP56, NOP58 and SNU13), FBL catalyzes ribose 2′-O methylation (2′-O-Me) in human rRNAs to accelerate ribosome biogenesis and internal ribosome entry site (IRES)-dependent translation [8]. Interestingly, results from both semi-quantitative RTL-P and quantitative RiboMeth-seq assays revealed a global downregulation of rRNA 2′-O-Me in EZH2-deficient PCa cells. Moreover, the IRES-dependent translation of a series of prostate oncogenes, such as XIAP, FGF2, IGF1R and MYC, was dramatically inhibited upon EZH2 depletion. Further mechanistic research by Yi et al. showed that EZH2 could recruit FBL and NOP56 together to construct a protein trimer. As a result, EZH2 facilitates box C/D snoRNP assembly by strengthening the FBL-NOP56 interaction, which serves as a crucial step during box C/D snoRNP biogenesis [9].

Since the aforementioned results indicated a significant contribution of EZH2 in translational control, Yi et al. next conducted Ribo-seq along with RNA-seq to map the altered patterns of ribosome-protected fragments and mRNA transcripts in EZH2-deficient PCa cells. The resulting data confirmed that EZH2 modulates gene expression at both transcriptional and translational levels, and verified that FBL is responsible for the EZH2-mediated translational regulation. Taking XIAP as an example, Yi et al. proved that the capability to interact with FBL and NOP56, but not its methyltransferase activity, is indispensable for EZH2 to promote IRES-dependent translation. Finally, Yi et al. demonstrated that both FBL and NOP56 function as crucial downstream effectors of EZH2, thus account for the EZH2-driven tumorigenesis of PCa. Taken together, this study uncovers a novel non-canonical role of EZH2 to orchestrate transcriptional regulation and translational control in PCa.

In the more recent report, Yi et al. further discovered an additional noncanonical role of EZH2 in co-activation of transcription [7]. CDCA8, a subunit of the chromosomal passenger complex (CPC) which plays an important role in mitosis, was found to be upregulated in PCa cells to potentiate the tumorigenic capacity. Remarkably, a significant positive correlation between EZH2 and CDCA8 was observed in PCa, indicating a potential regulatory relationship. To uncover the mechanism by which EZH2 promotes CDCA8 expression, Yi et al. firstly hypothesized that EZH2 may promote CDCA8 expression by downregulating the microRNAs (miRNAs) targeting CDCA8 [10]. To confirm this hypothesis, they performed small RNA-seq in control and EZH2-deficient PCa cells to define the EZH2-modulated miRNAs and compared with the list of miRNAs which were predicted to target CDCA8 transcripts. As a consequence, let-7b, a potential tumor suppressor of PCa, was identified as the top candidate for verification. Yi et al. revealed that EZH2-induced H3K27me3 modification at the promoter of let-7b represses its expression and thus prevents the let-7b targeting CDCA8 transcripts from degradation. Surprisingly, silencing of let-7b failed to fully rescue the downregulation of CDCA8 in EZH2-deficient PCa cells, indicating the existence of additional pathways for EZH2 to mediate CDCA8 in PCa.

Next, by checking the RNA-seq data in EZH2-knockdown PCa cells, the authors noticed that E2F1, a transcription factor and master cell cycle regulator, displayed a sharp downregulation upon EZH2 suppression. E2F1 specifically binds to the promoter regions of all CPC members including CDCA8 to activate transcription. Previous studies suggested that EZH2 could recruit E2F1 to activate transcription at chromatin sites lacking H3K27me3 marks [11]. Here, Yi et al. further proved that EZH2 could enhance the self-activation of E2F1 by recruiting E2F1 to its own promoter in a methylation-independent manner, which in turn activates transcription of CDCA8 along with the other CPC subunits to accelerate PCa cell proliferation.

In summary, both articles by Yi et al. emphasized the significance of non-canonical functions of EZH2 during PCa development, which may provide novel insights into the advancement of EZH2-targeting strategies to treat PCa patients. In fact, a new wave has been ushed for the discovery of EZH2 inhibitors to eliminate both the catalytic and non-catalytic activities of EZH2 [12–14]. Will these newly developed EZH2 degraders be successfully applied in PCa therapy? Will additional non-canonical functions of EZH2 be characterized in the PCa model? Let’s eagerly wait and see.
